# Impact of Mastectomy on Body Image and Sexuality from a LGBTQ Perspective: A Narrative Review

**DOI:** 10.3390/jcm10040567

**Published:** 2021-02-03

**Authors:** Magdalena Skórzewska, Andrzej Kurylcio, Karol Rawicz-Pruszyński, Wachirabhorn Chumpia, Buabongkoj Punnanan, Sasiwan Jirapongvanich, Tianxiao Jiang, Jerzy Mielko

**Affiliations:** Department of Surgical Oncology, Medical University of Lublin, Radziwiłłowska 13 St., 20-080 Lublin, Poland; magdalenaskorzewska@uml.edu.pl (M.S.); andrzejkurylcio@uml.edu.pl (A.K.); wachirabhorn.chumpia@gmail.com (W.C.); pun.bbk93@gmail.com (B.P.); sasiwanjir@gmail.com (S.J.); jiangtx@gmail.com (T.J.); jerzymielko@uml.edu.pl (J.M.)

**Keywords:** sexuality, mastectomy, LGBTQ+, heterosexual

## Abstract

Although mastectomy could lead to a decrease in sexual performance among patients, only a handful of studies focused on the psychological and sexual behavioral aspects after the surgery. Research on post-mastectomy sexuality has focused mainly on female subjects but barely on lesbian, gay, bisexual, transgender, queer (LGBTQ), and male patients. This narrative review aimed to explore the importance of sexuality after mastectomy from a LGBTQ perspective. Each sexual minority group has been addressed individually. In general, sexual and gender minority breast cancer (BC) patients undergoing bilateral mastectomy expect a complex treatment plan in terms of physical and emotional outcomes. Bilateral mastectomy or top surgery for masculinization reasons was reported to be the most popular procedure among transmen, which resulted in a significant improvement in the quality of life. Heterosexual and lesbian female patients are willing to undergo mastectomy after repeated lumpectomies or to avoid radiation, despite potential post-operative somatic and quality-of-life complications. Transwomen would seek gender-affirming surgery to improve physical satisfaction and psychological well-being. There is not enough evidence for non-oncological reasons and consequences of mastectomy in gay men and cisgender heterosexual men. Establishing the awareness of the sexuality impact of mastectomy will allow the implementation of tailored perioperative psychological care.

## 1. Introduction

Each group of lesbian, gay, bisexual, transgender, queer (LGBTQ) patients has unique and specific healthcare needs [1]. However, when provided a nonjudgmental and empathetic environment, experience of psychological care disparities can be eliminated. Recently, “practice pointers” for developing a more inclusive service for LGBTQ+ patients have been introduced. The areas of good clinical practice include direct patient care, healthcare environment, improving the healthcare evidence base, and education [2].

Surgical management of the breast, mastectomy, in particular, has transformed from a radicalized procedure that left patients with severe morbidity to an elegant operation that delicately balances safety with reconstructive principles [3].

Mastectomy is most commonly performed procedure due to breast cancer (BC), one of the most common cancers in the world [4]. Additionally, mastectomy plays a crucial role as prophylaxis for BC among high-risk populations [5]. Although the surgical procedure could lead to a decrease in the sexual performance among patients [6], only a handful of studies focused on the psychological and sexual behavioral aspects after the operation [7]. The vast majority of research on post-mastectomy sexuality has focused mainly on female subjects but barely on LGBTQ and male patients [8,9]. Meanwhile, sexual and gender minority BC patients undergoing mastectomy expect a complex treatment plan in terms of physical and emotional outcomes.

Traditionally, BC is the most common indication for mastectomy [4]. However, among transgender men, bilateral mastectomy is the most commonly performed gender-affirming surgery [10]. Other indications for mastectomy include risk-reducing mastectomy [11], Cowden disease [12], and giant juvenile fibroadenomas [13].

Policy and programmatic efforts are required to reduce negative experiences and their health impact on sexual and gender minority adults [14]. Therefore, there is an ethical necessity to provide a specialized multidisciplinary team in terms of diagnosis, follow-up, and treatment in this group of patients [15].

According to recent systematic review, the majority of patients who underwent breast reconstruction after prophylactic mastectomy had a satisfactory quality of life, despite sexual problems including decrease in sexual sensations and problems in enjoyment of sex [16]. The procedure may improve self-esteem and body image-related quality of life [17].

By establishing the awareness of the sexuality impact of mastectomy, the implementation of a tailored treatment plan and perioperative psychological care will be possible.

To the best of our knowledge, there are no data available on the influence of radical breast surgery on sexuality in both BC and gender-affirming patients. Therefore, this narrative review aimed to explore the importance of sexuality after mastectomy from a gender-related perspective. Indications and consequences of mastectomy in each gender group were addressed individually.

## 2. Materials and Methods

Two investigators (M.S., K.R.P.) performed a search for relevant literatures using the MEDLINE, EMBASE, and Scopus database. The search strategy included the following keywords and medical subject heading (MeSH) terms: “LGBTQ” OR “Lesbian” OR “Gay” OR “Bisexual” OR “Transgender” OR “Queer” OR “Questioning” OR “Transmen” OR “Transwomen” OR “Heterosexual” OR “Homosexual” OR “Cisgender” OR “Gender” OR “Sexual” OR “Minority” AND “Mastectomy” OR “Breast surgery” OR “Breast removal”. The search was limited to articles published in English. The studies included in this narrative review were chosen on the basis of the eligibility criteria.

## 3. Results

### 3.1. Transmen

As of March 2020, dedicated nationwide population data of transmen outside the U.S. was not found. Bilateral mastectomy or top surgery for masculinization reason was reported to be the most popular procedure among transmen, with 36% of the population reported having undergone surgery and 61% needing a future mastectomy [10]. Additionally, recent data suggest a year-over-year increase in mastectomy cases in transmen [18]. Much of the bodily dysphoria and social difficulties for transmen relate to their feminine breasts [19]. Despite the hesitance toward genital surgery, most of the transmen seem to plan to undergo (subcutaneous) mastectomy [20].

Top surgery is frequently described as a mastectomy with male chest reconstruction, which includes contouring the chest into a masculine shape [21]. The procedure is guided by four main principles: removal of breast tissue and skin excess, reduction and positioning of the nipple–areola complex, elimination of the inframammary fold, and minimizing chest wall scars.

Mastectomy was perceived diversely among unique gender populations. It caused a negative psychological impact, especially in women [22], including lesbians, due to a fear of desexualization [6]. On the contrary, mastectomy was shown to be an advantageous procedure that enables transmen to claim their place in society [23], have easier social interaction [24], and gain improvement in body satisfaction, which led to self-esteem development and higher quality of life [17]. Besides a surgical aspect, one year of testosterone therapy positively showed an increase of libido and masturbation in transmen after sexual reassignment surgery [25]. Gender-affirming surgery allows transmen patients to reach a high level of satisfaction and improved quality of life [26]. Mastectomy is, therefore, a favorable procedure that mostly has an overall positive impact on sexuality in transmen. Nevertheless, underrepresentation and discrimination of transmen [27], along with insufficient data involving the population, are calls to raise awareness of gender and sexuality variety in the healthcare aspect for the best interest in this group of patients. Noteworthy, transgender men who have not undergone breast reduction surgery should be screened for BC as cisgender women [10].

### 3.2. Lesbian

The available literature mostly focuses on the comparison between lesbian and heterosexual women in terms of response to BC screening, diagnosis, treatment, and follow-up. In general, sexual minority women with BC are similar to sexual minority women without cancer with respect to healthful behaviors, anxiety, depression, and quality of life [28]. Lesbian BC patients seem to be less interested in body appearance than heterosexual BC patients [8,29]. In addition, a survey among lesbian-specific posts in an online support forum showed rejection of the reconstruction trend [30]. However, a more recent study suggested that the consequences of losing sensation over the areola area and reduced sexual response were considered acceptable in those who decided not to undertake a reconstruction [31]. Some lesbian patients decided to reconstruct because of a desire to feel “whole” and “normal” [32]. The considerations of lesbians who decided for or against reconstruction after mastectomy are rooted in a value system that prioritizes body strength, survival, and physical functioning over outward appearance or normative beauty standard. Sexual minotrity patients who chose reconstruction experienced difficulties and regrets, whereas patients without reconstruction adjusted well after time [28].

Noteworthy, there is a mutual influence on quality of life between sexual minority BC survivors and their caregivers [33]. Better collection of sexual orientation and racial identity data in medical records is crucial for building the evidence base to understand disparities for those at the intersection of marginalized racial and sexual identities, as shown in a recent study on BC screening and care in Black sexual minority women [34]. Further prospective studies to establish an optimal psychological outcome in lesbian patients undergoing a mastectomy are, therefore, warranted.

### 3.3. Gay Men

Some heterosexual cisgender men have different demands than other cisgender men. Despite a growing number of gay men population [35], there is no gender-classified data collection of mastectomy cases. In general, gay men are 70–100 times less vulnerable to BC than female patients [36]. Nevertheless, an application of gender-classified data collection in future mastectomy cases as well as post-mastectomy on the sexuality of gay men patient is a necessity.

### 3.4. Transwomen

Transwomen would seek gender-affirming surgery [37], which can improve physical satisfaction and psychological well-being [38]. As a part of the gender-affirming procedure, hormone therapy was prescribed for most transwomen [39]. Despite the higher risk to develop BC, there is no data collection of mastectomy and sexuality of transwomen after mastectomy. Moreover, the existing data on BC cases in the transwomen population are rare [40]. On the other hand, further prospective studies will allow recognizing psychological outcomes in transwomen BC patients.

### 3.5. Cisgender Heterosexual Women

Mastectomy is one of the most common surgical procedure for BC in women, and research showed that it could lead to an impact on females both physically and psychologically [41]. In a prospective controlled study of 149 female BC patients, results showed that those who received the mastectomy reported problems in sexual dysfunctions by decreased sexual desire, arousal, and orgasm compared to before receiving the surgery [6]. Yaghjyan et al. showed that there was a significant decrease in estrogen level after mastectomy [42], which is one of the most critical factors influencing female sexuality [43]. A recent study showed that women who underwent mastectomy lost self-esteem of their physical characteristics, which leads to psychological distress [44,45,46], while body image issues could directly lead to low libido [47]. On the other hand, the investigation by Pakistani Women Post-Mastectomy reported that mastectomy had not affected their physical relationship with their spouse and did not have any concerns regarding sexuality after mastectomy [48]. Combined brief psychosexual intervention and counseling after a mastectomy is necessary to help post-mastectomy females in body image self-acceptance and perceived partner acceptance positively [49]. Excellent social support [50] and strong married relationships can improve the quality of sexual life after mastectomy [9].

### 3.6. Cisgender Heterosexual Men

Unsurprisingly, there is generally restricted information concerning the psychological outcomes of male BC treatment [51]. Brain et al. conducted a cross-sectional survey on 161 male BC cases, where only 1% of depression and 6% of the anxiety were clinically treatable, which is considerably lower than that reported in female BC [52]. Andrykowski [53] used the data of male BC from the US 2009 Behavioral Risk Factor Surveillance System (BRFSS) survey and contrasted them with 198 disease-free control samples. The results showed that male BC patients had reduced activity, higher risk of obesity, poorer life satisfaction, and worse general health. Research conducted by Trovão and Serefoglu showed that emotional disorders such as depression and anxiety have a significant relationship with decreasing sexuality (desire, arousal, orgasm, and ejaculation) in men due to altered neurotransmitter levels or connectivity [54]. However, there is no evidence shown that there is an increased incidence of psychological or sexual disorders after mastectomy among male BC. Numerous questions remain concerning the causes, outcomes, and treatment of BC in men. Further research is required to address gaps in information relating to the care of male BC patients and survivors [7].

### 3.7. Future Perspectives?

There is clear evidence that mastectomy had a negative effect psychologically in female and male patients with BC. Resources in the lesbian population suggested statistically unclear psychological impact after the surgery. Only in the transmen population superlative mental health improvement was reported. Studies were able to prove that mastectomy affects negatively on sexuality among female patients [6] as a result of decreased hormone level [42,43]. Due to a negative effect on mental health, couples’ counseling after a mastectomy is highly recommended [49]. On the other hand, for LGBTQ and male patients, there is not enough evidence to link the negative psychological impact with sexual dysfunction. However, several studies suggest that mastectomy affects this group of patients psychologically. Therefore, despite solely proper post-surgical physical care, healthcare providers should consider the implementation of tailored, gender-classified psychological care. For instance, there is no gender classification for transmen. Notwithstanding their female biological bodies, their gender identity and needs are different than women, lesbian, and bisexual women populations. Hence, their perception of mastectomy is distinct. In the lesbian and bisexual women population, breast surgery reminded them of their previous disease [32] and few patients after bilateral mastectomy revealed adverse sexuality problems with their partners [31]. Furthermore, in the male population, there is evidence suggesting that emotional disorders could happen among male BCs [53], and it has been proven that such disorders among male BC patients are less treatable than among female patients [52]. This finding implies that unique post-operative care for each gender identity is demanded. As men are a minor group of breast cancer patients and gender-classified data collections for LGBTQ are scarce, studies regarding their cases were not able to establish a direct relationship between mastectomy and sexuality [9]. Therefore, this short narrative is a call for diverse gender classification in data collection systems and more research into sexuality among LGBTQ and men breast cancer patients after mastectomy.

## 4. Conclusions

In terms of sexuality, mastectomy affects each gender differently. The majority of women reported a decrease in libido and a negative effect on psychological well-being. While the lesbian population reported having an unclear conclusion, transmen are the only population that reported high satisfaction and having an improvement in mental health as well as the quality of life after mastectomy. However, there is not enough evidence-based data for gay men and male patients to establish a direct link between mastectomy and sexuality. As a result, a data collection system with diverse gender classification and more research into sexuality after mastectomy among men and LGBTQ patients is needed.
